# Supplementary material for: Time-Series Sensory Analysis Provided Important TI Parameters for Masking the Beany Flavor of Soymilk
Source: Foods. 2023 Jul 19;12(14):2752. doi: 10.3390/foods12142752 (PMC10379375; doi:10.3390/foods12142752)
Supplement: Supplementary file 1 [file foods-12-02752-s001.zip › Suppl Figure_PDF.pdf]

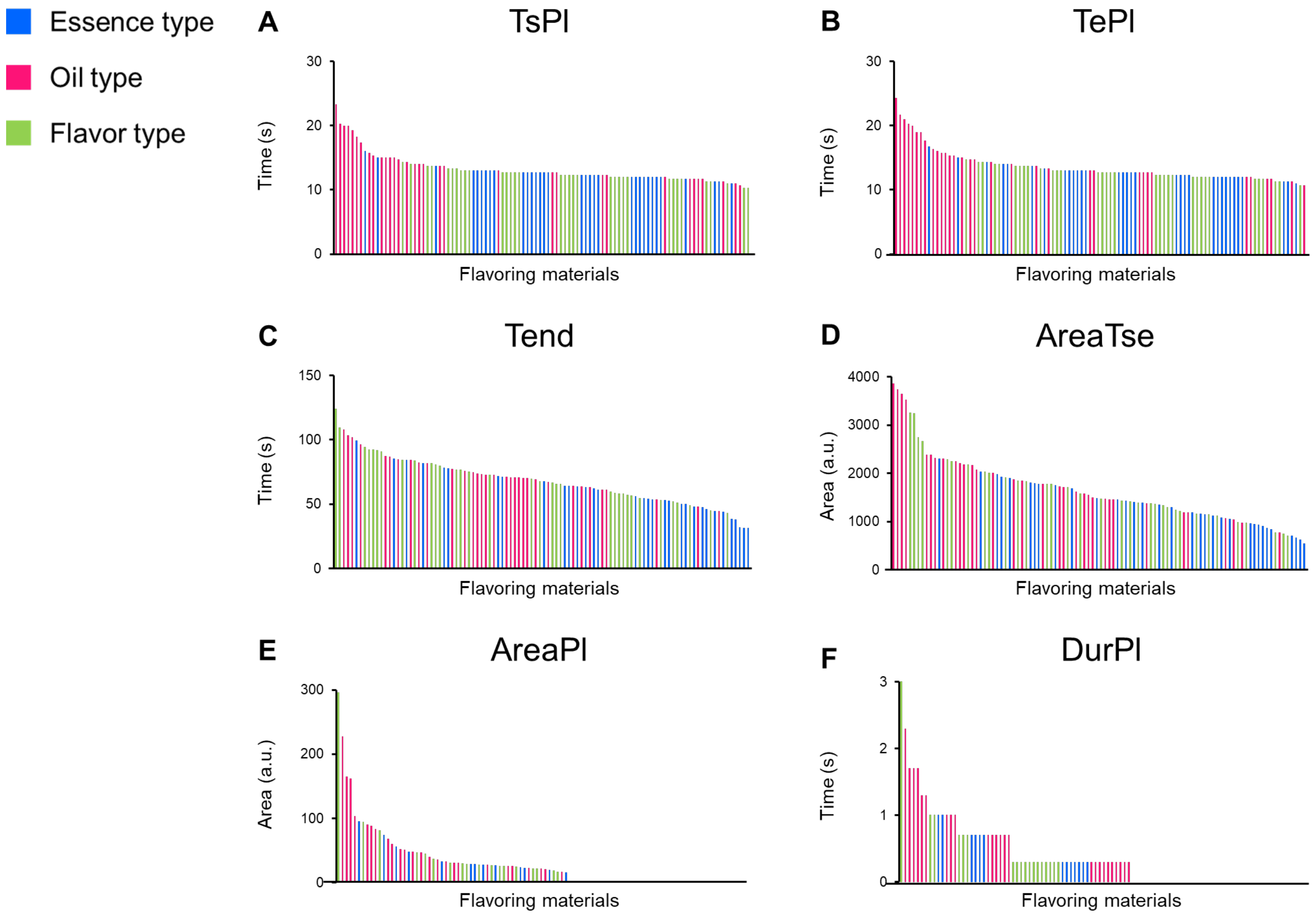

Figure S1: Six TI parameter values of the food-flavoring materials. The data values of the 100 flavor materials were arranged in descending order from left to right. The six TI parameters are as follows: (A) TsPl, (B) TePl, (C) Tend, (D) AreaTse, (E) AreaPl, and (F) DurPl. The colors of the bars indicate the material types: blue for essence type, magenta for oil type, and light green for flavor type. a.u. (arbitrary unit).
