# Supplementary material for: Time-Series Sensory Analysis Provided Important TI Parameters for Masking the Beany Flavor of Soymilk
Source: Foods. 2023 Jul 19;12(14):2752. doi: 10.3390/foods12142752 (PMC10379375; doi:10.3390/foods12142752)
Supplement: Supplementary file 1 [file foods-12-02752-s001.zip › Suppl Table.pdf]

Table S1. List of the 100 food-flavoring materials.

No.	Essence type	Notation	Origin	No.	Oil type	Notation	Origin	No.	Flavor type	Notation	Origin
1	Apple essence	Apple_e	artificial	34	Apple oil	Apple_o	artificial	67	Apricot flavor	Apricot_f	artificial
2	Apricot essence	Apricot_e	artificial	35	Almond oil	Almond_o	artificial	68	Baked sweet potato flavor	Baked sweet potato_f	artificial
3	Banana essence	Banana_e	artificial	36	Banana oil	Banana_o	artificial	69	Brown sugar flavor	Brown sugar_f	artificial
4	Blueberry essence	Blueberry_e	artificial	37	Blueberry oil	Blueberry_o	artificial	70	Chocolate flavor	Chocolate_f	artificial
5	Brown sugar essence	Brown sugar_e	artificial	38	Caramel oil	Caramel_o	artificial	71	Cocoa flavor	Cocoa_f	artificial
6	Caramel essence	Caramel_e	artificial	39	Cheese oil	Cheese_o	artificial	72	Coconut flavor	Coconut_f	artificial
7	Cheese essence	Cheese_e	artificial	40	Cherry oil	Cherry_o	artificial	73	Coffee flavor	Coffee_f	artificial
8	Cherry tree essence	Cherry tree_e	artificial	41	Cinnamon oil	Cinnamon_o	artificial	74	Cola flavor	Cola_f	artificial
9	Chestnut essence	Chestnut_e	artificial	42	Coconut oil	Coconut_o	artificial	75	Custard flavor	Custard_f	artificial
10	Chocolate essence	Chocolate_e	artificial	43	Cola oil	Cola_o	artificial	76	Framboise flavor	Framboise_f	artificial
11	Cinnamon essence	Cinnamon_e	artificial	44	European pear oil	European pear_o	artificial	77	Herb flavor	Herb_f	artificial
12	Cocoa essence	Cocoa_e	artificial	45	Guava oil	Guava_o	artificial	78	Honey flavor	Honey_f	artificial
13	Cranberry essence	Cranberry_e	artificial	46	Hazelnut oil	Hazelnut_o	artificial	79	Kabosu flavor	Kabosu_f	artificial
14	Grapefruit essence	Grapefruit_e	artificial	47	Kiwifruit oil	Kiwifruit_o	artificial	80	Kyoho grape flavor	Kyoho grape_f	artificial
15	Honey essence	Honey_e	artificial	48	Liche oil	Liche_o	artificial	81	Lemon flavor	Lemon_f	artificial
16	Kiwifruit essence	Kiwifruit_e	artificial	49	Mango oil	Mango_o	artificial	82	Liche flavor	Liche_f	artificial
17	Kyoho grape essence	Kyoho grape_e	artificial	50	Melon oil	Melon_o	artificial	83	Maple flavor	Maple_f	artificial
18	Lemon essence	Lemon_e	artificial	51	Muscat oil	Muscat_o	artificial	84	Matcha flavor	Matcha_f	artificial
19	Liche essence	Liche_e	artificial	52	Orange essential oil	Orange essential_o	natural	85	Melon flavor	Melon_f	artificial
20	Lime essence	Lime_e	artificial	53	Orange oil	Orange_o	artificial	86	Mixed fruit flavor	Mixed fruit_f	artificial
21	Mango essence	Mango_e	artificial	54	Peach oil	Peach_o	artificial	87	Muscat flavor	Muscat_f	artificial
22	Matcha essence	Matcha_e	artificial	55	Peppermint oil	Peppermint_o	artificial	88	Passion fruit flavor	Passionfruit_f	artificial
23	Melon essence	Melon_e	artificial	56	Pineapple oil	Pineapple_o	artificial	89	Peach flavor	Peach_f	artificial
24	Muscat essence	Muscat_e	artificial	57	Plum oil	Plum_o	artificial	90	Peanut flavor	Peanut_f	artificial
25	Orange essence	Orange_e	artificial	58	Raspberry oil	Raspberry_o	artificial	91	Peppermint flavor	Peppermint_f	artificial
26	Pineapple essence	Pineapple_e	artificial	59	Rose oil	Rose_o	artificial	92	Pineapple flavor	Pineapple_f	artificial
27	Raspberry essence	Raspberry_e	artificial	60	Satsuma mandarin essential oil	Satsuma mandarin essential_o	natural	93	Plum flavor	Plum_f	artificial
28	Rose essence	Rose_e	artificial	61	Soda pop oil	Soda pop_o	artificial	94	Roasted green tea flavor	Roasted green tea_f	artificial
29	Spearmint essence	Spearmint_e	artificial	62	Strawberry oil	Strawberry_o	artificial	95	Spearmint flavor	Spearmint_f	artificial
30	Strawberry essence	Strawberry_e	artificial	63	Tea oil	Tea_o	artificial	96	Sudachi flavor	Sudachi_f	artificial
31	Tea essence	Tea_e	artificial	64	Vanilla oil	Vanilla_o	artificial	97	Vanilla flavor	Vanilla_f	artificial
32	Vanilla essence	Vanilla_e	artificial	65	Yogurt oil	Yogurt_o	artificial	98	White peach flavor	White peach_f	artificial
33	White peach essence	White peach_e	artificial	66	Yuzu essential oil	Yuzu essential_o	natural	99	Yogurt flavor	Yogurt_f	artificial
								100	Yuzu flavor	Yuzu_f	artificial

The names of the materials were denoted as flavor name_type (e, o, or f) (e.g., apricot essence, almond oil, and brown sugar flavor were denoted as apricot_e, almond_o, and brown sugar_f, respectively). The food-flavoring materials were classified as artificial, artificially blended; natural, extracted from natural products.

Table S2. Masking scores and TI profiles of the flavor materials.

No.	Material name	Masking score	Imax	Tstart	Tend	TsPl	TePl	DurPl	DurInc	DurDec	SIMInc	SIMDec	AreaTse	AreaInc	AreaDec	AreaPl
1	Cinnamon_o	9.9	82.00	2.0	63.3	11.7	11.7	0.0	9.7	51.7	27.87	6.69	1381	334	1047	0
2	Peppermint_o	9.9	99.00	2.0	64.0	11.7	13.3	1.7	9.7	50.7	44.62	4.05	2248	411	1672	165
3	Orange essential_o	9.6	97.00	2.0	102.0	20.0	21.7	1.7	18.0	80.3	19.39	4.14	3650	1016	2472	162
4	Herb_f	9.5	98.67	2.0	92.0	10.3	13.3	3.0	8.3	78.7	17.63	10.18	3246	436	2514	296
5	Pineapple_o	9.3	75.33	2.0	82.3	12.7	12.7	0.0	10.7	69.7	15.99	3.28	2075	312	1763	0
6	Rose_o	9.3	97.67	2.0	108.0	15.3	17.7	2.3	13.3	90.3	16.58	5.02	3738	597	2913	228
7	Yuzu essential_o	9.3	78.67	2.0	73.3	20.3	21.0	0.7	18.3	52.3	6.04	4.67	2004	644	1307	52
8	Coffee_f	9.2	70.33	2.3	91.0	13.3	14.0	0.7	11.0	77.0	18.08	3.23	2183	357	1779	47
9	Rose_e	9.2	60.67	2.0	53.0	11.3	11.3	0.0	9.3	41.7	21.93	5.24	943	230	713	0
10	Cheese_o	9.1	91.67	2.0	71.3	11.7	12.0	0.3	9.7	59.3	21.93	6.75	1880	405	1445	31
11	Pineapple_f	9.0	80.33	2.0	43.0	11.3	11.7	0.3	9.3	31.3	31.17	11.21	989	332	630	27
12	Banana_e	9.0	78.67	2.0	84.3	12.0	12.0	0.0	10.0	72.3	19.44	3.04	2034	351	1684	0
13	Caramel_e	9.0	56.00	2.0	38.0	12.3	12.3	0.0	10.3	25.7	13.85	5.94	702	218	484	0
14	Cheese_e	9.0	76.00	2.0	31.3	11.3	11.3	0.0	9.3	20.0	21.16	8.43	848	296	552	0
15	Cherry_o	9.0	99.00	2.0	87.0	15.7	16.0	0.3	13.7	71.0	21.80	3.19	3521	735	2754	33
16	Yogurt_o	8.9	69.00	2.0	70.7	13.7	13.7	0.0	11.7	57.0	16.39	3.34	1549	387	1162	0
17	Plum_f	8.8	83.67	2.0	109.7	11.7	12.0	0.3	9.7	97.7	18.97	3.81	2742	360	2355	28
18	Liche_f	8.8	90.33	2.0	66.0	11.0	11.3	0.3	9.0	54.7	25.76	5.93	1854	340	1484	30
19	Strawberry_e	8.7	66.67	2.0	54.7	12.3	12.3	0.0	10.3	42.3	15.09	4.84	1127	313	814	0
20	Brown sugar_e	8.7	59.33	2.0	85.3	12.0	12.0	0.0	10.0	73.3	17.61	3.66	1426	213	1213	0
21	Matcha_e	8.7	73.00	2.0	99.7	12.0	12.0	0.0	10.0	87.7	19.22	3.15	2301	294	2007	0
22	Caramel_o	8.7	68.00	2.0	69.3	11.3	12.7	1.3	9.3	56.7	15.77	5.15	1462	272	1100	91
23	Hazelnut_o	8.7	72.00	2.0	44.7	10.7	10.7	0.0	8.7	34.0	26.46	5.35	976	241	734	0
24	Guava_o	8.7	65.67	2.0	70.3	12.3	12.7	0.3	10.3	57.7	11.92	2.70	1464	257	1184	22
25	Melon_f	8.5	93.00	2.0	84.3	13.0	13.0	0.0	11.0	71.3	16.20	8.38	2013	472	1541	0
26	Roasted green tea_f	8.5	85.00	2.3	67.7	13.7	13.7	0.0	11.3	54.0	25.40	7.50	1784	478	1306	0
27	Almond_o	8.5	85.00	1.7	70.7	13.7	14.0	0.3	12.0	56.7	22.73	2.74	2190	497	1665	28
28	Yogurt_f	8.4	92.00	2.0	76.7	14.3	14.7	0.3	12.3	62.0	16.30	5.14	2291	527	1733	31
29	Tea_e	8.4	82.00	2.0	72.0	12.7	13.0	0.3	10.7	59.0	24.71	5.90	1791	373	1391	27
30	Satsuma mandarin essential_o	8.4	89.00	2.0	103.3	23.3	24.3	1.0	21.3	79.0	8.15	2.27	3867	919	2859	89
31	Matcha_f	8.2	72.67	2.3	81.7	11.7	11.7	0.0	9.3	70.0	18.17	3.90	1473	265	1208	0
32	Honey_e	8.2	55.00	2.3	44.0	11.0	11.0	0.0	8.7	33.0	18.58	7.46	623	209	414	0
33	Mango_e	8.1	74.00	1.5	78.5	13.0	14.0	1.0	11.5	64.5	19.61	4.33	1749	363	1312	74
34	Blueberry_e	8.1	63.00	2.0	48.3	12.0	12.0	0.0	10.0	36.3	21.23	5.60	963	269	695	0
35	Cola_o	8.1	60.00	2.0	70.3	14.0	14.7	0.7	12.0	55.7	8.96	3.03	1581	315	1226	40
36	Orange_o	8.0	76.67	2.3	84.3	19.3	20.0	0.7	17.0	64.3	19.30	3.51	2313	765	1496	51
37	Spearmint_f	7.9	81.33	1.7	69.7	12.0	12.0	0.0	10.3	57.7	22.51	5.00	1586	344	1243	0

38	Muscat_f	7.9	81.00	2.0	80.0	13.0	14.0	1.0	11.0	66.0	13.91	3.79	2030	436	1514	81
39	Cola_f	7.9	60.67	2.0	72.7	13.7	13.7	0.0	11.7	59.0	14.74	3.42	1218	303	915	0
40	Orange_e	7.9	49.33	2.0	47.7	16.0	16.7	0.7	14.0	31.0	7.29	2.94	913	286	594	33
41	Grapefruit_e	7.9	83.33	2.0	64.3	12.7	12.7	0.0	10.7	51.7	19.73	2.97	1981	356	1625	0
42	Melon_o	7.9	70.00	2.0	96.7	15.0	15.3	0.3	13.0	81.3	14.55	3.34	2299	428	1848	23
43	Kyoho grape_e	7.8	70.67	2.0	71.3	12.3	12.7	0.3	10.3	58.7	15.82	5.28	1404	306	1075	24
44	Mango_o	7.8	93.00	2.0	82.0	15.0	15.3	0.3	13.0	66.7	17.98	6.16	2380	595	1754	31
45	Coconut_o	7.8	79.00	2.0	85.0	20.0	20.3	0.3	18.0	64.7	18.41	4.14	2206	776	1404	26
46	Kiwifruit_o	7.8	72.33	2.0	63.7	14.0	14.7	0.7	12.0	49.0	20.81	2.98	1732	416	1268	48
47	Spearmint_e	7.7	95.67	2.0	53.7	13.0	14.0	1.0	11.0	39.7	11.48	5.47	1896	489	1311	96
48	Vanilla_o	7.7	79.33	2.3	61.0	14.3	14.3	0.0	12.0	46.7	19.12	4.48	1849	443	1406	0
49	Kiwifruit_e	7.5	72.33	2.0	77.7	15.0	15.0	0.0	13.0	62.7	13.82	3.28	1785	413	1372	0
50	Peach_o	7.5	50.67	2.3	61.0	12.7	13.0	0.3	10.3	48.0	10.34	2.34	1076	207	853	17
51	Tea_o	7.5	64.33	2.0	73.7	12.3	12.7	0.3	10.3	61.0	16.98	7.12	1197	273	903	21
52	Vanilla_f	7.3	74.00	2.0	75.3	12.3	12.3	0.0	10.3	63.0	18.99	4.33	1717	339	1377	0
53	Apricot_e	7.3	84.33	1.7	63.7	12.7	13.0	0.3	11.0	50.7	25.18	4.85	1691	392	1271	28
54	White peach_f	7.2	57.00	2.0	45.3	12.0	12.0	0.0	10.0	33.3	12.07	6.34	742	228	515	0
55	Muscat_e	7.1	87.00	2.3	82.0	12.0	12.0	0.0	9.7	70.0	31.48	6.67	1928	370	1558	0
56	Banana_o	7.1	62.67	2.0	75.0	17.3	19.0	1.7	15.3	56.0	10.38	4.64	1616	482	1030	104
57	Maple_f	7.0	95.00	2.0	124.0	13.3	14.3	1.0	11.3	109.7	22.40	2.93	3257	563	2600	95
58	Cranberry_e	7.0	87.00	2.0	38.7	13.0	13.0	0.0	11.0	25.7	27.70	12.72	1080	387	693	0
59	Raspberry_e	7.0	64.33	2.0	64.3	12.3	12.3	0.0	10.3	52.0	20.75	3.25	1395	289	1106	0
60	Melon_e	6.9	82.67	1.7	62.3	12.0	12.0	0.0	10.3	50.3	16.35	13.44	1194	397	797	0
61	Vanilla_e	6.9	43.67	2.7	61.3	13.0	13.7	0.7	10.3	47.7	10.87	1.70	1051	188	835	29
62	Muscat_o	6.9	79.33	2.0	73.0	11.0	11.3	0.3	9.0	61.7	28.45	3.98	1718	275	1416	26
63	Coconut_f	6.8	77.00	2.7	92.3	12.7	13.0	0.3	10.0	79.3	21.48	2.68	2257	319	1912	26
64	Apricot_f	6.8	76.00	2.0	58.0	12.0	12.3	0.3	10.0	45.7	37.40	4.93	1362	327	1010	25
65	Strawberry_o	6.8	54.00	2.0	53.7	15.0	15.7	0.7	13.0	38.0	7.98	2.53	1197	303	858	36
66	Yuzu_f	6.7	75.00	2.3	52.3	12.3	12.3	0.0	10.0	40.0	18.30	4.79	1251	290	960	0
67	Peppermint_f	6.7	66.33	2.3	54.7	14.0	14.3	0.3	11.7	40.3	15.63	3.36	1397	318	1056	22
68	Honey_f	6.6	50.33	2.0	51.0	10.3	10.7	0.3	8.3	40.3	19.42	3.56	702	147	539	17
69	Liche_e	6.6	86.67	2.0	50.3	12.7	13.0	0.3	10.7	37.3	24.36	16.19	1299	435	836	29
70	Pineapple_e	6.5	59.00	2.3	32.0	13.0	13.3	0.3	10.7	18.7	14.56	6.20	663	264	380	20
71	Peanut_f	6.4	85.67	2.0	77.0	11.7	12.0	0.3	9.7	65.0	24.91	3.60	1911	377	1505	29
72	Cinnamon_e	6.4	72.33	2.0	44.7	13.0	13.0	0.0	11.0	31.7	19.46	4.72	1148	316	832	0
73	European pear_o	6.3	67.67	2.0	87.3	14.0	15.0	1.0	12.0	72.3	19.99	2.69	2176	386	1722	68
74	Plum_o	6.1	63.33	2.0	75.7	15.0	16.3	1.3	13.0	59.3	10.18	3.01	1785	355	1346	84
75	Soda pop_o	5.9	59.67	2.0	77.3	14.7	15.7	1.0	12.7	61.7	10.82	2.97	1496	267	1169	60
76	Peach_f	5.8	56.00	2.3	57.0	13.0	13.7	0.7	10.7	43.3	12.52	2.71	1170	253	880	37
77	Passionfruit_f	5.8	63.00	2.0	94.3	12.7	12.7	0.0	10.7	81.7	18.34	1.96	1836	270	1567	0
78	Chestnut_e	5.8	71.33	2.0	56.0	13.7	14.3	0.7	11.7	41.7	15.90	4.18	1460	339	1074	48
79	Liche_o	5.7	68.67	2.0	71.0	13.0	13.0	0.0	11.0	58.0	20.17	5.23	1468	322	1146	0
80	Lime_e	5.4	52.33	2.0	46.0	12.3	12.3	0.0	10.3	33.7	15.59	2.82	870	224	646	0
81	Cocoa_f	5.2	75.33	2.0	83.7	12.7	12.7	0.0	10.7	71.0	16.28	2.72	1775	312	1463	0
82	Baked sweet potato_f	5.0	65.67	2.0	58.7	12.0	12.0	0.0	10.0	46.7	24.94	4.63	1120	281	839	0

83	Chocolate_f	4.9	65.67	2.3	66.7	12.0	12.3	0.3	9.7	54.3	19.26	3.66	1333	226	1085	22
84	Custard_f	4.8	67.67	2.3	92.3	12.3	13.0	0.7	10.0	79.3	18.43	2.17	2670	286	2339	45
85	Blueberry_o	4.8	55.00	2.0	67.3	12.0	12.0	0.0	10.0	55.3	11.92	4.11	1039	210	829	0
86	Chocolate_e	4.6	67.33	2.0	63.3	12.7	12.7	0.0	10.7	50.7	25.96	2.58	1492	317	1174	0
87	Mixed fruit_f	4.4	57.00	2.0	53.0	11.7	11.7	0.0	9.7	41.3	15.14	3.30	975	214	761	0
88	Brown sugar_f	4.4	49.67	2.0	49.3	12.7	12.7	0.0	10.7	36.7	15.45	4.13	775	210	565	0
89	Cherry tree_e	4.3	63.33	2.0	54.3	12.0	12.0	0.0	10.0	42.3	16.49	6.82	934	273	661	0
90	Framboise_f	4.1	76.67	2.0	56.7	12.3	12.3	0.0	10.3	44.3	15.60	11.10	1144	337	806	0
91	Kyoho grape_f	4.1	57.67	2.0	80.7	12.3	12.7	0.3	10.3	68.0	14.37	5.74	1304	247	1037	19
92	Apple_e	4.1	68.33	2.0	50.0	12.7	13.0	0.3	10.7	37.0	10.16	5.02	1160	326	811	23
93	Lemon_f	4.0	77.33	2.0	59.7	12.7	12.7	0.0	10.7	47.0	20.67	4.15	1380	353	1026	0
94	Lemon_e	3.7	87.33	2.0	52.7	12.7	12.7	0.0	10.7	40.0	18.36	7.14	1347	353	994	0
95	White peach_e	3.7	49.33	2.0	31.7	11.7	12.0	0.3	9.7	19.7	15.06	6.13	551	183	352	16
96	Sudachi_f	3.6	83.67	2.0	58.3	11.3	11.3	0.0	9.3	47.0	27.93	5.16	1438	298	1140	0
97	Kabosu_f	3.6	77.67	2.0	65.7	13.3	13.7	0.3	11.3	52.0	13.61	4.42	1419	314	1079	26
98	Apple_o	3.6	70.67	2.0	72.7	18.3	19.0	0.7	16.3	53.7	10.30	1.52	2381	558	1775	47
99	Raspberry_o	3.5	59.00	2.3	48.0	11.7	11.7	0.0	9.3	36.3	18.01	6.45	775	221	554	0
100	Cocoa_e	1.2	84.00	1.7	67.7	12.0	12.7	0.7	10.3	55.0	31.04	4.72	1815	367	1392	56

Table S3. List of the 100 food-flavoring materials arranged in descending order for each of the eight TI parameters.

A) Tstart		B) Imax		C) AreaInc		D) DurInc		E) SIMInc		F) AreaDec		G) DurDec		H) SIMDec	
Coconut_f	2.7	Cherry_o	99.0	Maple_f	109.7	Satsuma mandarin essential_o	21.3	Peppermint_o	44.62	Rose_o	2913	Maple_f	109.7	Liche_e	16.19
Vanilla_e	2.7	Peppermint_o	99.0	Plum_f	97.7	Yuzu essential_o	18.3	Apricot_f	37.40	Satsuma mandarin essential_o	2859	Plum_f	97.7	Melon_e	13.44
Yuzu_f	2.3	Herb_f	98.7	Rose_o	90.3	Coconut_o	18.0	Muscat_e	31.48	Cherry_o	2754	Rose_o	90.3	Cranberry_e	12.72
Roasted green tea_f	2.3	Rose_o	97.7	Matcha_e	87.7	Orange essential_o	18.0	Pineapple_f	31.17	Maple_f	2600	Matcha_e	87.7	Pineapple_f	11.21
Peach_f	2.3	Orange essential_o	97.0	Passionfruit_f	81.7	Orange_o	17.0	Cocoa_e	31.04	Herb_f	2514	Passionfruit_f	81.7	Framboise_f	11.10
Chocolate_f	2.3	Spearmint_e	95.7	Melon_o	81.3	Apple_o	16.3	Muscat_o	28.45	Orange essential_o	2472	Melon_o	81.3	Herb_f	10.18
Peppermint_f	2.3	Maple_f	95.0	Orange essential_o	80.3	Banana_o	15.3	Sudachi_f	27.93	Plum_f	2355	Orange essential_o	80.3	Cheese_e	8.43
Coffee_f	2.3	Melon_f	93.0	Coconut_f	79.3	Orange_e	14.0	Cinnamon_o	27.87	Custard_f	2339	Coconut_f	79.3	Melon_f	8.38
Custard_f	2.3	Mango_o	93.0	Custard_f	79.3	Cherry_o	13.7	Cranberry_e	27.70	Matcha_e	2007	Custard_f	79.3	Roasted green tea_f	7.50
Matcha_f	2.3	Yogurt_f	92.0	Satsuma mandarin essential_o	79.0	Rose_o	13.3	Hazelnut_o	26.46	Coconut_f	1912	Satsuma mandarin essential_o	79.0	Honey_e	7.46
Pineapple_e	2.3	Cheese_o	91.7	Herb_f	78.7	Kiwifruit_e	13.0	Chocolate_e	25.96	Melon_o	1848	Herb_f	78.7	Lemon_e	7.14
Muscat_e	2.3	Liche_f	90.3	Coffee_f	77.0	Melon_o	13.0	Liche_f	25.76	Coffee_f	1779	Coffee_f	77.0	Tea_o	7.12
Honey_e	2.3	Satsuma mandarin essential_o	89.0	Brown sugar_e	73.3	Mango_o	13.0	Roasted green tea_f	25.40	Apple_o	1775	Brown sugar_e	73.3	Cherry tree_e	6.82
Peach_o	2.3	Lemon_e	87.3	Banana_e	72.3	Plum_o	13.0	Apricot_e	25.18	Pineapple_o	1763	Banana_e	72.3	Cheese_o	6.75
Vanilla_o	2.3	Cranberry_e	87.0	European pear_o	72.3	Strawberry_o	13.0	Baked sweet potato_f	24.94	Mango_o	1754	European pear_o	72.3	Cinnamon_o	6.69
Orange_o	2.3	Muscat_e	87.0	Melon_f	71.3	Soda pop_o	12.7	Peanut_f	24.91	Yogurt_f	1733	Melon_f	71.3	Muscat_e	6.67
Raspberry_o	2.3	Liche_e	86.7	Cocoa_f	71.0	Yogurt_f	12.3	Tea_e	24.71	European pear_o	1722	Cocoa_f	71.0	Raspberry_o	6.45
Melon_f	2.0	Peanut_f	85.7	Cherry_o	71.0	European pear_o	12.0	Liche_e	24.36	Banana_e	1684	Cherry_o	71.0	White peach_f	6.34
Pineapple_f	2.0	Roasted green tea_f	85.0	Matcha_f	70.0	Vanilla_o	12.0	Almond_o	22.73	Peppermint_o	1672	Matcha_f	70.0	Pineapple_e	6.20
Lemon_f	2.0	Almond_o	85.0	Muscat_e	70.0	Almond_o	12.0	Spearmint_f	22.51	Almond_o	1665	Muscat_e	70.0	Mango_o	6.16
Sudachi_f	2.0	Apricot_e	84.3	Pineapple_o	69.7	Kiwifruit_o	12.0	Maple_f	22.40	Grapefruit_e	1625	Pineapple_o	69.7	White peach_e	6.13
White peach_f	2.0	Cocoa_e	84.0	Kyoho grape_f	68.0	Cola_o	12.0	Rose_e	21.93	Passionfruit_f	1567	Kyoho grape_f	68.0	Caramel_e	5.94
Kabosu_f	2.0	Sudachi_f	83.7	Mango_o	66.7	Peppermint_f	11.7	Cheese_o	21.93	Muscat_e	1558	Mango_o	66.7	Liche_f	5.93
Herb_f	2.0	Plum_f	83.7	Muscat_f	66.0	Cola_f	11.7	Cherry_o	21.80	Melon_f	1541	Muscat_f	66.0	Tea_e	5.90
Plum_f	2.0	Grapefruit_e	83.3	Peanut_f	65.0	Chestnut_e	11.7	Coconut_f	21.48	Muscat_f	1514	Peanut_f	65.0	Kyoho grape_f	5.74
Liche_f	2.0	Melon_e	82.7	Coconut_o	64.7	Yogurt_o	11.7	Blueberry_e	21.23	Peanut_f	1505	Coconut_o	64.7	Blueberry_e	5.60
Framboise_f	2.0	Tea_e	82.0	Mango_e	64.5	Mango_e	11.5	Cheese_e	21.16	Orange_o	1496	Mango_e	64.5	Spearmint_e	5.47
Yogurt_f	2.0	Cinnamon_o	82.0	Orange_o	64.3	Kabosu_f	11.3	Kiwifruit_o	20.81	Liche_f	1484	Orange_o	64.3	Hazelnut_o	5.35
Baked sweet potato_f	2.0	Spearmint_f	81.3	Vanilla_f	63.0	Roasted green tea_f	11.3	Raspberry_e	20.75	Cocoa_f	1463	Vanilla_f	63.0	Kyoho grape_e	5.28
Vanilla_f	2.0	Muscat_f	81.0	Kiwifruit_e	62.7	Maple_f	11.3	Lemon_f	20.67	Cheese_o	1445	Kiwifruit_e	62.7	Rose_e	5.24
Cocoa_f	2.0	Pineapple_f	80.3	Yogurt_f	62.0	Melon_f	11.0	Liche_o	20.17	Muscat_o	1416	Yogurt_f	62.0	Liche_o	5.23
Muscat_f	2.0	Vanilla_o	79.3	Muscat_o	61.7	Muscat_f	11.0	European pear_o	19.99	Vanilla_o	1406	Muscat_o	61.7	Sudachi_f	5.16
Peanut_f	2.0	Muscat_o	79.3	Soda pop_o	61.7	Coffee_f	11.0	Grapefruit_e	19.73	Coconut_o	1404	Soda pop_o	61.7	Caramel_o	5.15

Apricot_f	2.0	Coconut_o	79.0	Tea_o	61.0	Spearmint_e	11.0	Mango_e	19.61	Cocoa_e	1392	Tea_o	61.0	Yogurt_f	5.14
Maple_f	2.0	Banana_e	78.7	Cheese_o	59.3	Cranberry_e	11.0	Cinnamon_e	19.46	Tea_e	1391	Cheese_o	59.3	Apple_e	5.02
Cola_f	2.0	Yuzu essential_o	78.7	Plum_o	59.3	Apricot_e	11.0	Banana_e	19.44	Vanilla_f	1377	Plum_o	59.3	Rose_o	5.02
Mixed fruit_f	2.0	Kabosu_f	77.7	Cola_f	59.0	Cinnamon_e	11.0	Honey_f	19.42	Kiwifruit_e	1372	Cola_f	59.0	Spearmint_f	5.00
Kyoho grape_f	2.0	Lemon_f	77.3	Tea_e	59.0	Liche_o	11.0	Orange essential_o	19.39	Plum_o	1346	Tea_e	59.0	Apricot_f	4.93
Honey_f	2.0	Coconut_f	77.0	Kyoho grape_e	58.7	Lemon_f	10.7	Orange_o	19.30	Mango_e	1312	Kyoho grape_e	58.7	Apricot_e	4.85
Brown sugar_f	2.0	Framboise_f	76.7	Liche_o	58.0	Cocoa_f	10.7	Chocolate_f	19.26	Spearmint_e	1311	Liche_o	58.0	Strawberry_e	4.84
Passionfruit_f	2.0	Orange_o	76.7	Spearmint_f	57.7	Peach_f	10.7	Matcha_e	19.22	Yuzu essential_o	1307	Spearmint_f	57.7	Yuzu_f	4.79
Lemon_e	2.0	Apricot_f	76.0	Guava_o	57.7	Brown sugar_f	10.7	Vanilla_o	19.12	Roasted green tea_f	1306	Guava_o	57.7	Cocoa_e	4.72
Banana_e	2.0	Cheese_e	76.0	Yogurt_o	57.0	Passionfruit_f	10.7	Vanilla_f	18.99	Apricot_e	1271	Yogurt_o	57.0	Cinnamon_e	4.72
Orange_e	2.0	Cocoa_f	75.3	Caramel_o	56.7	Lemon_e	10.7	Plum_f	18.97	Kiwifruit_o	1268	Caramel_o	56.7	Yuzu essential_o	4.67
White peach_e	2.0	Pineapple_o	75.3	Almond_o	56.7	Liche_e	10.7	Honey_e	18.58	Spearmint_f	1243	Almond_o	56.7	Banana_o	4.64
Kyoho grape_e	2.0	Yuzu_f	75.0	Banana_o	56.0	Pineapple_e	10.7	Custard_f	18.43	Cola_o	1226	Banana_o	56.0	Baked sweet potato_f	4.63
Liche_e	2.0	Vanilla_f	74.0	Cola_o	55.7	Apple_e	10.7	Coconut_o	18.41	Brown sugar_e	1213	Cola_o	55.7	Vanilla_o	4.48
Spearmint_e	2.0	Mango_e	74.0	Blueberry_o	55.3	Chocolate_e	10.7	Lemon_e	18.36	Matcha_f	1208	Blueberry_o	55.3	Kabosu_f	4.42
Cranberry_e	2.0	Matcha_e	73.0	Cocoa_e	55.0	Tea_e	10.7	Passionfruit_f	18.34	Guava_o	1184	Cocoa_e	55.0	Vanilla_f	4.33
Strawberry_e	2.0	Matcha_f	72.7	Liche_f	54.7	Grapefruit_e	10.7	Yuzu_f	18.30	Chocolate_e	1174	Liche_f	54.7	Mango_e	4.33
Kiwifruit_e	2.0	Kiwifruit_e	72.3	Chocolate_f	54.3	Pineapple_o	10.7	Matcha_f	18.17	Soda pop_o	1169	Chocolate_f	54.3	Chestnut_e	4.18
Apple_e	2.0	Cinnamon_e	72.3	Roasted green tea_f	54.0	Spearmint_f	10.3	Coffee_f	18.08	Yogurt_o	1162	Roasted green tea_f	54.0	Lemon_f	4.15
Caramel_e	2.0	Kiwifruit_o	72.3	Apple_o	53.7	Framboise_f	10.3	Raspberry_o	18.01	Liche_o	1146	Apple_o	53.7	Coconut_o	4.14
Blueberry_e	2.0	Hazelnut_o	72.0	Yuzu essential_o	52.3	Vanilla_f	10.3	Mango_o	17.98	Sudachi_f	1140	Yuzu essential_o	52.3	Orange essential_o	4.14
Raspberry_e	2.0	Chestnut_e	71.3	Kabosu_f	52.0	Kyoho grape_f	10.3	Herb_f	17.63	Raspberry_e	1106	Kabosu_f	52.0	Brown sugar_f	4.13
Chocolate_e	2.0	Kyoho grape_e	70.7	Raspberry_e	52.0	Melon_e	10.3	Brown sugar_e	17.61	Caramel_o	1100	Raspberry_e	52.0	Blueberry_o	4.11
Tea_e	2.0	Apple_o	70.7	Grapefruit_e	51.7	Kyoho grape_e	10.3	Tea_o	16.98	Chocolate_f	1085	Grapefruit_e	51.7	Peppermint_o	4.05
Chestnut_e	2.0	Coffee_f	70.3	Cinnamon_o	51.7	Strawberry_e	10.3	Rose_o	16.58	Kabosu_f	1079	Cinnamon_o	51.7	Muscat_o	3.98
Cheese_e	2.0	Melon_o	70.0	Chocolate_e	50.7	Caramel_e	10.3	Cherry tree_e	16.49	Kyoho grape_e	1075	Chocolate_e	50.7	Matcha_f	3.90
Grapefruit_e	2.0	Yogurt_o	69.0	Apricot_e	50.7	Raspberry_e	10.3	Yogurt_o	16.39	Chestnut_e	1074	Apricot_e	50.7	Plum_f	3.81
Brown sugar_e	2.0	Liche_o	68.7	Peppermint_o	50.7	Cocoa_e	10.3	Melon_e	16.35	Peppermint_f	1056	Peppermint_o	50.7	Muscat_f	3.79
Cinnamon_e	2.0	Apple_e	68.3	Melon_e	50.3	Lime_e	10.3	Yogurt_f	16.30	Cinnamon_o	1047	Melon_e	50.3	Chocolate_f	3.66
Lime_e	2.0	Caramel_o	68.0	Kiwifruit_o	49.0	Vanilla_e	10.3	Cocoa_f	16.28	Kyoho grape_f	1037	Kiwifruit_o	49.0	Brown sugar_e	3.66
Rose_e	2.0	Custard_f	67.7	Peach_o	48.0	Peach_o	10.3	Melon_f	16.20	Banana_o	1030	Peach_o	48.0	Peanut_f	3.60
Cherry tree_e	2.0	European pear_o	67.7	Vanilla_e	47.7	Guava_o	10.3	Pineapple_o	15.99	Lemon_f	1026	Vanilla_e	47.7	Honey_f	3.56
Matcha_e	2.0	Chocolate_e	67.3	Lemon_f	47.0	Tea_o	10.3	Chestnut_e	15.90	Apricot_f	1010	Lemon_f	47.0	Orange_o	3.51
Melon_o	2.0	Strawberry_e	66.7	Sudachi_f	47.0	White peach_f	10.0	Kyoho grape_e	15.82	Lemon_e	994	Sudachi_f	47.0	Cola_f	3.42
Liche_o	2.0	Peppermint_f	66.3	Baked sweet potato_f	46.7	Yuzu_f	10.0	Caramel_o	15.77	Yuzu_f	960	Baked sweet potato_f	46.7	Peppermint_f	3.36
European pear_o	2.0	Baked sweet potato_f	65.7	Vanilla_o	46.7	Coconut_f	10.0	Peppermint_f	15.63	Cola_f	915	Vanilla_o	46.7	Melon_o	3.34
Caramel_o	2.0	Chocolate_f	65.7	Apricot_f	45.7	Baked sweet potato_f	10.0	Framboise_f	15.60	Tea_o	903	Apricot_f	45.7	Yogurt_o	3.34
Mango_o	2.0	Guava_o	65.7	Framboise_f	44.3	Apricot_f	10.0	Lime_e	15.59	Peach_f	880	Framboise_f	44.3	Mixed fruit_f	3.30
Coconut_o	2.0	Raspberry_e	64.3	Peach_f	43.3	Custard_f	10.0	Brown sugar_f	15.45	Strawberry_o	858	Peach_f	43.3	Kiwifruit_e	3.28
Cheese_o	2.0	Tea_o	64.3	Strawberry_e	42.3	Banana_e	10.0	Mixed fruit_f	15.14	Peach_o	853	Strawberry_e	42.3	Pineapple_o	3.28
Plum_o	2.0	Cherry tree_e	63.3	Cherry tree_e	42.3	Blueberry_e	10.0	Strawberry_e	15.09	Baked sweet potato_f	839	Cherry tree_e	42.3	Raspberry_e	3.25
Strawberry_o	2.0	Plum_o	63.3	Chestnut_e	41.7	Brown sugar_e	10.0	White peach_e	15.06	Liche_e	836	Chestnut_e	41.7	Coffee_f	3.23
Banana_o	2.0	Passionfruit_f	63.0	Rose_e	41.7	Cherry tree_e	10.0	Cola_f	14.74	Vanilla_e	835	Rose_e	41.7	Cherry_o	3.19

Apple_o	2.0	Blueberry_e	63.0	Mixed fruit_f	41.3	Matcha_e	10.0	Pineapple_e	14.56	Cinnamon_e	832	Mixed fruit_f	41.3	Matcha_e	3.15
Pineapple_o	2.0	Banana_o	62.7	Peppermint_f	40.3	Blueberry_o	10.0	Melon_o	14.55	Blueberry_o	829	Peppermint_f	40.3	Banana_e	3.04
Muscat_o	2.0	Cola_f	60.7	Honey_f	40.3	Plum_f	9.7	Kyoho grape_f	14.37	Strawberry_e	814	Honey_f	40.3	Cola_o	3.03
Cherry_o	2.0	Rose_e	60.7	Yuzu_f	40.0	Peanut_f	9.7	Muscat_f	13.91	Apple_e	811	Yuzu_f	40.0	Plum_o	3.01
Kiwifruit_o	2.0	Cola_o	60.0	Lemon_e	40.0	Chocolate_f	9.7	Caramel_e	13.85	Framboise_f	806	Lemon_e	40.0	Kiwifruit_o	2.98
Cola_o	2.0	Soda pop_o	59.7	Spearmint_e	39.7	Mixed fruit_f	9.7	Kiwifruit_e	13.82	Melon_e	797	Spearmint_e	39.7	Grapefruit_e	2.97
Blueberry_o	2.0	Brown sugar_e	59.3	Strawberry_o	38.0	White peach_e	9.7	Kabosu_f	13.61	Mixed fruit_f	761	Strawberry_o	38.0	Soda pop_o	2.97
Hazelnut_o	2.0	Pineapple_e	59.0	Liche_e	37.3	Muscat_e	9.7	Peach_f	12.52	Hazelnut_o	734	Liche_e	37.3	Orange_e	2.94
Cinnamon_o	2.0	Raspberry_o	59.0	Apple_e	37.0	Cheese_o	9.7	White peach_f	12.07	Rose_e	713	Apple_e	37.0	Maple_f	2.93
Yogurt_o	2.0	Kyoho grape_f	57.7	Brown sugar_f	36.7	Cinnamon_o	9.7	Blueberry_o	11.92	Blueberry_e	695	Brown sugar_f	36.7	Lime_e	2.82
Peppermint_o	2.0	White peach_f	57.0	Blueberry_e	36.3	Peppermint_o	9.7	Guava_o	11.92	Cranberry_e	693	Blueberry_e	36.3	Almond_o	2.74
Guava_o	2.0	Mixed fruit_f	57.0	Raspberry_o	36.3	Pineapple_f	9.3	Spearmint_e	11.48	Cherry tree_e	661	Raspberry_o	36.3	Cocoa_f	2.72
Tea_o	2.0	Peach_f	56.0	Hazelnut_o	34.0	Sudachi_f	9.3	Vanilla_e	10.87	Lime_e	646	Hazelnut_o	34.0	Peach_f	2.71
Rose_o	2.0	Caramel_e	56.0	Lime_e	33.7	Matcha_f	9.3	Soda pop_o	10.82	Pineapple_f	630	Lime_e	33.7	Guava_o	2.70
Soda pop_o	2.0	Honey_e	55.0	White peach_f	33.3	Cheese_e	9.3	Banana_o	10.38	Orange_e	594	White peach_f	33.3	Pear_o	2.69
Yuzu essential_o	2.0	Blueberry_o	55.0	Honey_e	33.0	Rose_e	9.3	Peach_o	10.34	Brown sugar_f	565	Honey_e	33.0	Coconut_f	2.68
Orange essential_o	2.0	Strawberry_o	54.0	Cinnamon_e	31.7	Caramel_o	9.3	Apple_o	10.30	Raspberry_o	554	Cinnamon_e	31.7	Chocolate_e	2.58
Satsuma mandarin essential_o	2.0	Lime_e	52.3	Pineapple_f	31.3	Raspberry_o	9.3	Plum_o	10.18	Cheese_e	552	Pineapple_f	31.3	Strawberry_o	2.53
Spearmint_f	1.7	Peach_o	50.7	Orange_e	31.0	Liche_f	9.0	Apple_e	10.16	Honey_f	539	Orange_e	31.0	Peach_o	2.34
Melon_e	1.7	Honey_f	50.3	Cranberry_e	25.7	Muscat_o	9.0	Cola_o	8.96	White peach_f	515	Cranberry_e	25.7	Satsuma mandarin essential_o	2.27
Cocoa_e	1.7	Brown sugar_f	49.7	Caramel_e	25.7	Honey_e	8.7	Satsuma mandarin essential_o	8.15	Caramel_e	484	Caramel_e	25.7	Custard_f	2.17
Apricot_e	1.7	Orange_e	49.3	Cheese_e	20.0	Hazelnut_o	8.7	Strawberry_o	7.98	Honey_e	414	Cheese_e	20.0	Passionfruit_f	1.96
Almond_o	1.7	White peach_e	49.3	White peach_e	19.7	Herb_f	8.3	Orange_e	7.29	Pineapple_e	380	White peach_e	19.7	Vanilla_e	1.70
Mango_e	1.5	Vanilla_e	43.7	Pineapple_e	18.7	Honey_f	8.3	Yuzu essential_o	6.04	White peach_e	352	Pineapple_e	18.7	Apple_o	1.52

Table S4. Materials belonging to clusters 0 and 1.

Materials	masking score	cluster	Materials	masking score	cluster	Materials	masking score	cluster
Peppermint_o	9.9	0	Cinnamon_o	9.9	1	Peach_f	5.8	1
Orange essential_o	9.6	0	Rose_e	9.2	1	Chestnut_e	5.8	1
Pineapple_o	9.3	0	Pineapple_f	9.0	1	Liche_o	5.7	1
Yuzu essential_o	9.3	0	Caramel_e	9.0	1	Lime_e	5.4	1
Coffee_f	9.2	0	Cheese_e	9.0	1	Baked sweet potato_f	5.0	1
Cheese_o	9.1	0	Yogurt_o	8.9	1	Chocolate_f	4.9	1
Liche_f	8.8	0	Strawberry_e	8.7	1	Blueberry_o	4.8	1
Matcha_e	8.7	0	Brown sugar_e	8.7	1	Chocolate_e	4.6	1
Melon_f	8.5	0	Caramel_o	8.7	1	Mixed fruit_f	4.4	1
Almond_o	8.5	0	Hazelnut_o	8.7	1	Brown sugar_f	4.4	1
Yogurt_f	8.4	0	Guava_o	8.7	1	Cherry tree_e	4.3	1
Tea_e	8.4	0	Roasted green tea_f	8.5	1	Framboise_f	4.1	1
Mango_e	8.1	0	Matcha_f	8.2	1	Kyoho grape_f	4.1	1
Cola_o	8.1	0	Honey_e	8.2	1	Apple_e	4.1	1
Orange_o	8.0	0	Blueberry_e	8.1	1	Lemon_f	4.0	1
Spearmint_f	7.9	0	Cola_f	7.9	1	Lemon_e	3.7	1
Muscat_f	7.9	0	Orange_e	7.9	1	White peach_e	3.7	1
Grapefruit_e	7.9	0	Kyoho grape_e	7.8	1	Sudachi_f	3.6	1
Melon_o	7.9	0	Kiwifruit_e	7.5	1	Kabosu_f	3.6	1
Mango_o	7.8	0	Peach_o	7.5	1	Raspberry_o	3.5	1
Coconut_o	7.8	0	Tea_o	7.5	1			
Kiwifruit_o	7.8	0	White peach_f	7.2	1			
Spearmint_e	7.7	0	Banana_o	7.1	1			
Vanilla_o	7.7	0	Cranberry_e	7.0	1			
Vanilla_f	7.3	0	Raspberry_e	7.0	1			
Apricot_e	7.3	0	Melon_e	6.9	1			
Muscat_e	7.1	0	Vanilla_e	6.9	1			
Muscat_o	6.9	0	Apricot_f	6.8	1			
Coconut_f	6.8	0	Strawberry_o	6.8	1			
Peanut_f	6.4	0	Yuzu_f	6.7	1			
European pear_o	6.3	0	Peppermint_f	6.7	1			
Plum_o	6.1	0	Honey_f	6.6	1			
Passionfruit_f	5.8	0	Liche_e	6.6	1			
Cocoa_f	5.2	0	Pineapple_e	6.5	1			
Apple_o	3.6	0	Cinnamon_e	6.4	1			
Cocoa_e	1.2	0	Soda pop_o	5.9	1			
